# Thermal and Physical Characterization of PEG Phase Change Materials Enhanced by Carbon-Based Nanoparticles

**DOI:** 10.3390/nano10061168

**Published:** 2020-06-15

**Authors:** David Cabaleiro, Samah Hamze, Jacek Fal, Marco A. Marcos, Patrice Estellé, Gaweł Żyła

**Affiliations:** 1 Departamento de Física Aplicada, Facultade de Ciencias, Universidade de Vigo, 36310 Vigo, Spain; mmarcosm@uvigo.es; 2 Laboratoire de Génie Civil et Génie Mécanique, LGCGM, Université Rennes, 35000 Rennes, France; samah.hamze@univ-rennes1.fr; 3 Department of Experimental Physics, Rzeszow University of Technology, 35-959 Rzeszow, Poland; jacekfal@prz.edu.pl

**Keywords:** NePCM, PEG400, carbon black, graphite, nano-diamond, solid-liquid phase change, thermal conductivity, surface tension, dynamic viscosity, density

## Abstract

This paper presents the preparation and thermal/physical characterization of phase change materials (PCMs) based on poly(ethylene glycol) 400 g·mol^−1^ and nano-enhanced by either carbon black (CB), a raw graphite/diamond nanomixture (G/D-r), a purified graphite/diamond nanomixture (G/D-p) or nano-Diamond nanopowders with purity grades of 87% or 97% (nD87 and nD97, respectively). Differential scanning calorimetry and oscillatory rheology experiments were used to provide an insight into the thermal and mechanical changes taking place during solid-liquid phase transitions of the carbon-based suspensions. PEG400-based samples loaded with 1.0 wt.% of raw graphite/diamond nanomixture (G/D-r) exhibited the lowest sub-cooling effect (with a reduction of ~2 K regarding neat PEG400). The influences that the type of carbon-based nanoadditive and nanoparticle loading (0.50 and 1.0 wt.%) have on dynamic viscosity, thermal conductivity, density and surface tension were also investigated in the temperature range from 288 to 318 K. Non-linear rheological experiments showed that all dispersions exhibited a non-Newtonian pseudo-plastic behavior, which was more noticeable in the case of carbon black nanofluids at low shear rates. The highest enhancements in thermal conductivity were observed for graphite/diamond nanomixtures (3.3–3.6%), while nano-diamond suspensions showed the largest modifications in density (0.64–0.66%). Reductions in surface tension were measured for the two nano-diamond nanopowders (nD87 and nD97), while slight increases (within experimental uncertainties) were observed for dispersions prepared using the other three carbon-based nanopowders. Finally, a good agreement was observed between the experimental surface tension measurements performed using a Du Noüy ring tensiometer and a drop-shape analyzer.

## 1. Introduction

Energy consumption is increasing rapidly to meet the requirements of the growing world population and rising demand from industrialized countries [1,2]. This fact, together with the intensive use of energy sources from fossil fuels, has triggered several environmental issues [3,4]. Since this situation is expected to become much worse in the coming years, a large-scale transformation of current worldwide energy policies towards sustainability is essential [5]. Thermal energy storage, TES, can substantially contribute to such a transition towards a green energy system. Thus, by stocking thermal energy when available so that it can be released for utilization when necessary, it is possible to address the mismatches between demand and supply when recovering waste heat or harvesting renewable energy sources [6].

Materials can accumulate thermal energy in three main different ways: sensible heat (by using a temperature difference), latent heat (when undergoing a change in phase), and thermo-chemical (during a reversible reaction by breaking and forming molecular bonds) storage [7]. Latent TES technologies based on phase change materials, PCMs, exhibit large store capacities in a temperature range near to their phase change (charging and discharging processes present a nearly isothermal nature). Among different phase transitions, solid-liquid and solid-solid are particularly attractive since they provide high energy storage densities, something that, in turn, reduces the technological requirements of TES systems [8]. PCMs have been widely used in latent heat accumulation to improve the energy management in heat pumps [9,10], automotive [11], solar engineering [12,13], spacecraft [14,15] or heating, ventilation, and air conditioning facilities [16,17,18,19], for instance.

Over the last decades several materials have been proposed as PCMs and classified according to their temperature range of interest [8,11,20]. Polymeric compounds such as poly(ethylene glycol)s, PEGs, are very promising candidates owing to their relatively high latent heats, chemical/thermal stabilities, non-corrosiveness and suitable transition temperatures, which can be tuned by means of the polymer molecular mass [21,22,23]. In particular, the PEG400 used as base material in our investigation has a melting point of ~277–281 K, which is attractive for cold thermal storage [24]. However, as with many other organic materials, PEGs have rather low thermal conductivities (in the range of 0.2–0.3 W·m^−1^·K^−1^ [11,25,26]), which slow the thermal charging/discharging of accumulated heat and may hinder broad-scale practical utilization [27].

Traditional attempts to intensify the heat transfer performance in TES tanks mainly consisted of extending the heat exchange surface used to charge or discharge the stored energy [19,28,29]. A revision of previous investigations shows finned surfaces [30,31,32,33,34], multi-tubular systems [35] or lessing rings [36] with different configurations were used to achieve a more rapid response and efficient thermal management of stored heat. When it comes to poly(ethylene glycol)-based systems, Baygi et al. [34] investigated the thermal management of photovoltaic solar cells integrated with PEG1000 and extended fins. The results showed that, by embedding the PCM with fins, the efficiency of the system to convert solar energy into electricity enhanced by 8%. Similar investigations were recently conducted by Firoozzadeh et al. [31,32] using PEG600 as phase change material. In these last cases, a PCM-fin combination was also proven effective to control the temperature, and efficiency of electricity production enhanced by 2–3%.

Encapsulating the PCMs within a polymeric/inorganic/metallic shell [37,38,39] or impregnating them into fiber matrixes [40], porous materials [41,42] or foams [43,44] are other research techniques for heat transfer enhancement [45]. In particular, porous supports have sparked enormous interest because of their large, specific surface areas, high volume porosity and excellent temperature stability [46]. Although porous materials such as zeolites, diatom earth, gamma alumina or expanded perlite have also been considered as subtracts [47], most scientific effort regarding poly(ethylene glycol)s has been addressed to the development of supports based on silica, SiO_2_, [48,49,50,51] or carbon structures [52,53,54,55]. Thus, Feng et al. [53] prepared PEG100-based composites using active carbon and silica molecular sieves. Among the investigated form-stabilized PCMs, PEG/AC combinations containing 80 wt.% of poly(ethylene glycol) exhibited the largest latent heat, the least super-cooling and the better heat storage efficiency. Wang et al. [54] developed active carbon and mesoporous carbon supports (with different pore structures) to stabilize PEG6000 and observed that the hexagonal pore structure promoted the filling and impregnation of the mesoporous support with PEG molecules. More recently, Chen et al. [55] embedded PEG8000 in a highly graphitized 3D network to shape-stabilize the PCM and achieve superior thermal energy harvesting. Such nano-composites could be potentially attractive in electronics systems such as computers to buffer excessive rises in temperature. A thorough control of support porosity is a key issue to avoid a considerable reduction in the space to be occupied by the PCM, which may reduce the total energy storage density. Additionally, as reported by Feng et al. [53] and Wang et al. [46], a tiny porous size can lead to very low enthalpy (or even zero) due to the strong confinement effect on the mobility of PEG molecules.

Although effective at promoting heat transfer, the use of an ample quantity of fins/fillers/fibers usually undermines energy storage density and increases the cost of the storage systems [56]. For that reason, in those applications in which leakage is not a major concern and a shape stabilization or encapsulation can be avoided, heat transfer performance can be also enhanced by dispersing highly-conductive nano-sized particles [57]. This especial kind of latent media, also known as nano-enhanced phase change materials (NePCMs) or nano-PCMs [58,59,60], stems from the progresses made over the last decades in the development of the advanced heat transfer fluids called nanofluids [61]. Metal oxide [62,63,64], metallic [65,66,67,68,69] and carbon-based nanomaterials [70,71,72,73,74,75] have been used as thermal conductivity enhancers to improve the thermal conductivity of poly(ethylene glycols). In addition to improving the effective thermal conductivity of PCMs, the dispersion of nanoparticles has a second advantage: it promotes nucleation during solidification/recrystallization [76]. A better nucleation rate reduces the difference in temperature between melting and solidifying transitions (sub-cooling effect), and consequently, the temperature range the material needs to cover to reversely undergo solid-liquid phase change is also shorter [77].

He et al. [75] prepared graphene nanoplatelets/PEG4000 nano-composites with various contents of graphene and studied the improvements in the thermal and photo-thermal performance of the new NePCMs in comparison with the neat poly(ethylene glycol). A thermal conductivity enhancement of up to 146% and a reduction of only 6.3% in latent heat were obtained for a 2% mass concentration of graphene nanoplatelets, while photo-thermal conversion results indicated that the designed nano-composites were very promising for the application in solar energy conversion and storage. Wang et al. [72] prepared nano-enhanced phase change materials as dispersions of sodium dodecyl sulfate (SDS) functionalized multiwall carbon nanotubes (MWCNTs) in PEG8000 with various nanoparticle loadings (0.5–5 wt.%). Melting temperature and latent heat slightly decreased from 337 to 335 K and from 165 to 150 J·g^−1^ as MWCNT content increased from 0.5 to 5% in mass, respectively. Conversely, thermal conductivity rose with nanoparticle loading from 0.295 to 0.531 W·m^−1^·K^−1^.

Ranjbar et al. [78] investigated the stability and thermal conductivity of phase change materials based on either commercial paraffin wax, stearic acid or polyethylene glycol (with a melting point ~331–336 K) and containing either pristine or functionalized multi-walled carbon nanotubes. Stability results showed that functionalized MWCNT or SDBS-assisted MWCNT suspensions had higher stability in PEG8000 than pristine MWCNTs, while thermal conductivity measurements revealed that in liquid phase the addition of 1 wt.% of nanoparticles enhanced this property by 16.6% in comparison to neat PEG8000. Marcos et al. [67,71,73] prepared and characterized NePCMs based on poly(ethylene glycol) 400 and containing functionalized graphene nanoplatelets [73], multi-walled carbon nanotubes [71] or silver nanoparticles [67]. These energy storage media, envisaged for low-temperature applications, reached enhancements in thermal conductivity ranging from 3.9% (for the silver dispersion at 1.1 wt.% loading [67]) to 23% (in the case of the functionalized graphene at 0.5 wt.% [73]), while the sub-cooling effect reduced by 7.1% in the case of the silver/PEG400 sample with 1.1% of nanoparticles [67].

In addition to a high latent heat at a phase change temperature suitable for the intended use of the TES system and an appropriate thermal conductivity that ensures a rapid exchange of stored energy, other thermophysical properties such as dynamic viscosity, surface tension or volumetric behavior are also important. Thus, for example, reliable information regarding dynamic viscosity is necessary to estimate the pressure drop and pumping power in those cases in which work in the liquid phase requires a pumping system [56,77]. Likewise, surface tension is related to the crystallization ability of materials, whereas volumetric properties are required to determine volume changes during phase change or due to the effect of temperature [79,80]. Additionally, an accurate characterization of these physical and thermal properties is also necessary to describe melt convection heat transfer response by means of different non-dimensional ratios such as Nusselt, Rayleigh, Stefan, or Fourier numbers.

Marcos et al. [71] studied the flow behavior of PEG400-based dispersions loaded with 0.01–1.0 wt.% of multi-walled carbon nanotubes and observed that, except for 0.01% dispersion that was Newtonian, all NePCMs exhibited a shear-thinning behavior. Yapici et al. [62] studied the rheological behavior of TiO_2_ dispersions in PEG200 containing nanoparticle concentrations in the range 1–10 wt.%. Non-linear viscoelastic tests revealed a non-Newtonian pseudo-plastic behavior for TiO_2_ contents larger than 1%, while oscillatory analyses evidenced that highly loaded samples showed a gel-like structure. Zafarani-Moattar and Majdan-Cegincara [64] investigated the volumetric and (magneto)rheological properties for PEG400-based dispersions of Fe_3_O_4_ nanoparticles either bared or coated with oleic acid. In the studied range of volumetric concentrations, from 0.48–3.6%, samples showed a pseudo-plastic flow behavior. Additionally, obtained results from excess molar volume and isentropic compressibility analyses evidenced that oleic acid covalently binds to the surface of iron oxide nanoparticles. Qi et al. [23] designed PEG1000-based phase change materials shape-stabilized with graphene oxide and doped with graphene nanoplatelets as thermal enhancers. The authors completed their thermal study of the designed composites with an analysis of the mechanical properties during the phase change throughout oscillatory rheological tests.

In this study, different NePCMs were designed as dispersions of carbon black (CB), a raw graphite/diamond nanomixture (G/D-r), a purified graphite/diamond nanomixture (G/D-p) or a nano-Diamond nanopowder with purity grades of either 87% (nD87) or 97% (nD97) in poly(ethylene glycol) 400 g·mol^−1^ at two different nanoparticle loadings (0.5 and 1.0 wt.%). Various experimental techniques were carried out to investigate the thermal and physical properties of those materials. Solid-liquid phase transitions were determined by means of differential scanning calorimetry and oscillatory rheological experiments. In the temperature range from 288 to 318 K (in which PEG400 is in liquid phase), thermal conductivity was investigated using a transient hot wire instrument, surface tension was obtained using two experimental devices (a Du Noüy ring tensiometer and a drop-shape analyzer), dynamic viscosity was determined using a rotational rheometer, and the volumetric analyses were performed on a U-tube densimeter.

## 2. Materials and Methods

### 2.1. Materials and Nanofluid Preparation

Five different carbon-based nanomaterials, all them supplied by PlasmaChem GmbH (Berlin, Germany), were used as nanoadditives, viz., carbon black, CB; nano-diamonds purified with grade G, nD87; nano-diamonds purified with grade G01, nD97; a purified graphite/diamond nanomixture, G/D-p; and a raw graphite/diamond nanomixture, G/D-r. The average sizes and purities declared by the manufacturer are listed in Table 1.

Thorough size and morphological analyses of batches of these same five commercial nanopowders were recently carried out by Vallejo et al. [81]. According to the transmission electron microscopy (TEM) studies reported, nD87 and nD97 powders contain quasi-spherical nanoparticles with average sizes close in value to ~4 nm, indicated by the supplier. In addition, nearly spherical nanoparticles were observed in TEM micrographs of G/D-p and G/D-r nanoparticles. In G/D-p and G/D-r, it was also possible to distinguish two nanoparticle size populations: the tiniest ones corresponded to nano-diamonds, while the others (with slightly larger size and more frequently present) were graphite nanoparticles. Finally, TEM images of CB nanopowders showed nanoparticles with irregular shape and sizes of ~10–15 nm.

In this study, nanoparticle densities of 1.9, 3.1, 3.2, 2.52 and 2.54 g/cm^−3^ were considered for carbon back (CB) [82], nano-diamonds purified with grade G (nD87), nano-diamonds purified with grade G01 (nD97), purified graphite/diamond nanomixture, (G/D-p) [83] and raw graphite/diamond nanomixture (G/D-r) [83], respectively. The densities for the two nano-diamond mixtures were calculated as the weight average value between the densities of graphite (2.26 g/cm^−3^ [82]) and nano-diamonds (3.2 g·cm^−3^ [84]), considering the amount of nano-diamonds (87 and 97% in mass) and impurities (all assumed with the same density as graphite)

**Figure 1:**
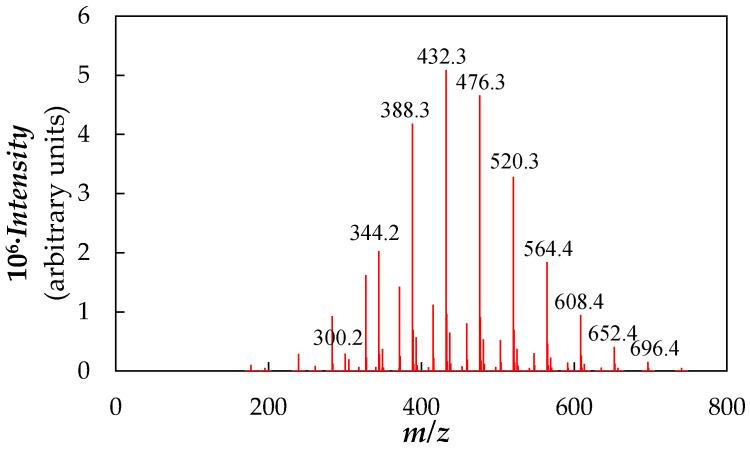
Positive ion electrospray ionization (ESI) mass spectra of neat PEG400.

The material used as base fluid was a poly(ethylene glycol) with a declared molecular mass of ~400 g·mol^−1^. As for the nanoparticles, the base fluid was used as supplied, without any further chemical purification or treatment. A high-resolution APEX Qe FT–ICR (Bruker Daltonics, Billerica, MA, USA) mass spectrometer based on electrospray ionization (ESI) was utilized to confirm the purity and molecular mass of the PEG400. Figure 1 shows a representative ESI mass spectrum obtained in positive ion mode.

Studied polymer molecules have an expected structure like HO–[CH_2_–CH_2_–O]_n_–H. Hence, all detected peaks in the mass spectrum can be attributed to poly(ethylene glycol) molecules mainly cationized with H^+^, NH_4_^+^, 2H^+^, Na^+^ or K^+^ with *n* = 4–14. The sample exhibits a uniform molecular mass with an average number of *M_n_* = 424.5 g·mol^−1^, an average value of *M_w_* = 443.1 g·mol^−1^ and a polydispersity index of *M_w_*/*M_n_* = 1.044. These values are closer in size to those reported by Marcos et al. [67,71,73] for other commercial PEG400.

Five different nanofluid sets, viz., CB/PEG400, nD87/PEG400, nD97/PEG400, G/D-p/PEG400 and G/D-r/PEG400, were prepared at 0.5 and 1.0% nanoparticle mass concentrations via a two-step method. Thus, the required amounts of nanopowder and base fluid necessary to obtain the predefined concentrations were weighted on an analytical balance Radwag AS 220/X (Radwag, Radom, Poland) with an accuracy of 1 × 10^−4^ g. After mechanically shaking the mixtures using a Genius 3 (IKA, Staufen, Germany) vortex system for 30 min, samples were sonicated for 200 min in an Emmi 60 HC ultrasound wave bath (EMAG, Moerfelden-Walldorf, Germany) working with a ultrasound power of 450 W and a frequency of 45 kHz [81]. Special care was taken to periodically replace the water of the ultrasonic bath and avoid sample overheating during sonication process.

### 2.2. Thermal and Physical Characterization

Melting and crystallization processes were characterized in terms of temperature and enthalpy by means of a Q2000 (TA Instruments, New Castle, WA, USA) differential scanning calorimetry, DSC, equipped with a refrigerated cooling system RSC90. Scans were performed in the temperature range from 223.15 to 313.15 K under nitrogen atmosphere (mole fraction purity higher than 0.99999) using ~10 μg of sample hermetically encapsulated in 20 μL T_zero_^TM^ aluminium pans. Before experiments, studied materials were held at a temperature of 313 K (more than 20 K above their melting transition) to erase the prior thermal history of the materials, and the first freeze-thaw cycle was disregarded. Estimated uncertainties are 0.3 K (with a repeatability of 0.1 K) for transition temperatures and 1.2 J·g^−1^ (with a repeatability of 0.7 J·g^−1^) for latent heats [85].

Rheological studies were carried out on a Malvern Kinexus Pro (Malvern Instruments Ltd., Worcestershire, UK) stress-controlled rheometer [86]. Temperature was established and controlled to within ±0.2 K using a Peltier system placed below the lower plate. Three different types of tests were carried out. First, the shear rate dependence of dynamic viscosity (also referred as flow curve) was studied at 4 isotherms in the range from 288.15 to 318.15 K (within which the PEG400 is in liquid phase) by means of non-linear viscoelastic tests. For such analyses, a cone-plate geometry with a rotating cone 60 mm in diameter, cone angle of 1° and truncation of 30 μm was coupled to the rheometer. Viscosity, *η*, values were collected in steady-state conditions at shear stresses logarithmically increasing from 0.8 to 200 Pa with at least 10 points per decimal. The experimental uncertainty of viscosity measurements using this experimental device is 4% [86]. Then, oscillatory strain sweeps were performed at 253.15 and 263.15 K (at which the PEG400 used as base fluid is in solid phase) as well as at 283.15 and 293.15 K (PEG400 is in liquid phase) in order to partially define the linear viscoelastic region. In such rheological oscillatory experiments the frequency was fixed at 1 Hz, while the strain amplitude was progressively increased up to ~0.1% when NePCMs were in solid phase (253.15 and 263.15 K) and up to ~1% when liquid (283.15 and 293.15 K). Finally, temperature sweeps were performed in the range between 253.15 and 293.15 K in cooling and heating mode using a constant scanning rate of 0.5 K·min^−1^ to ensure uniform temperature conditions throughout the sample. In these last assays, frequency was fixed at 1 Hz and strain was kept as low as 0.05% to guarantee linear viscoelastic behavior of the materials in the whole analyzed temperature range. Strain and temperature sweeps were performed using a plate-plate geometry of 40 mm in diameter and a constant gap of 1 mm, and sandpaper was glued to the upper plate to prevent slippage [87].

Thermal conductivity, *k*, was studied in the temperature range from 288.15 to 318.15 K (PEG400 is in liquid phase) by means of a THW-L2 thermal conductivity meter (Thermtest Inc., Fredericton, NB, Canada). This device is based on the transient short hot wire (THW) technique and allows direct *k* determinations of liquids in accordance with the ASTM D7896-14 standard test method [88]. The alumel wire used as probe has a length of 60 mm and a diameter of ~0.1 mm. The probe is vertically immersed in the sample, which completely surrounds the sensor, so that heat can freely diffuse in all directions. Both probe and sample are contained in a proper vessel, which is placed in a thermostatic bath to ensure uniform initial temperature. A low thermal power, 80 mW, and a short power input time, 1.5 s, were selected to avoid natural convection phenomena. The accuracy in thermal conductivity determinations with this device declared by the manufacturer is 5%. However, previous tests with water and ethylene glycol [89,90] show average deviations with literature [91] better than 3%. More details about this device and the followed experimental method can be found in a recent work by Banisharif et al. [89].

Densities, *ρ*, of neat PEG400 and carbon-based suspensions were experimentally determined at atmospheric pressure and in the temperature range from 288.15 to 313.15 K by means of a DMA 500 (Anton Paar, Graz, Autria) vibrating tube densimeter. Measurements with this device show an uncertainty of ±0.02 K in temperature and ±0.0005 g·cm^−3^ in density [92].

Following a previous investigation on ethylene glycol-based nanofluids prepared using various types of nitride nanoparticles recently published by Wanic et al. [93], two different methods were considered in the present work to determine the surface tension at the sample fluid/air interface, *σ* or SFT. A comparison of the results produced using the two methods will provide information on the consistency of the two techniques to study the surface tension of nanostructured samples. First, this property was studied for the neat PEG400 and nanofluids in the temperature range from 288 to 318 K by means of a DSA-30 (KRÜSS GmBH, Hamburg, Germany) drop-shape analyzer based on the pendant drop technique [94]. Sample temperature was controlled within ±0.1 K using a TC40 environmental chamber (also from Krüss GmbH). A needle with an outside diameter of 15 gauge (1.83 mm) was used to create drops with a volume of ~15–16 μL. The experimental uncertainty of measurements performed with the drop-shape analyzer was estimated to be less than 1% [95]. Surface tension measurements were also performed at 298 and 308 K for the base fluid and the highest concentration of each carbon nanofluid set using a PI–MT1A.KOM (Polon–Izot, Warsaw, Poland) surface tensiometer based on the Du Noüy ring method. In this case, determinations were performed using a ring with a radius of 9.691 mm and made of a platinum-iridium wire (0.203 mm in radius). Previous surface tension studies performed for substances of well-known SFT with this same Du Noüy tensiometer showed deviations within 1% [93]. Further details about the principles of both techniques and the followed experimental procedures can be found in Wanic et al. [93].

## 3. Results and Discussion

### 3.1. Solid-Liquid Phase Change

#### 3.1.1. Differential Scanning Calorimetry Studies

The thermal profiles of solid-liquid transitions were studied in the temperature range from 223.15 to 313.15 K using DSC at scan rates of 0.5 K·min^−1^. 

**Figure 2:**
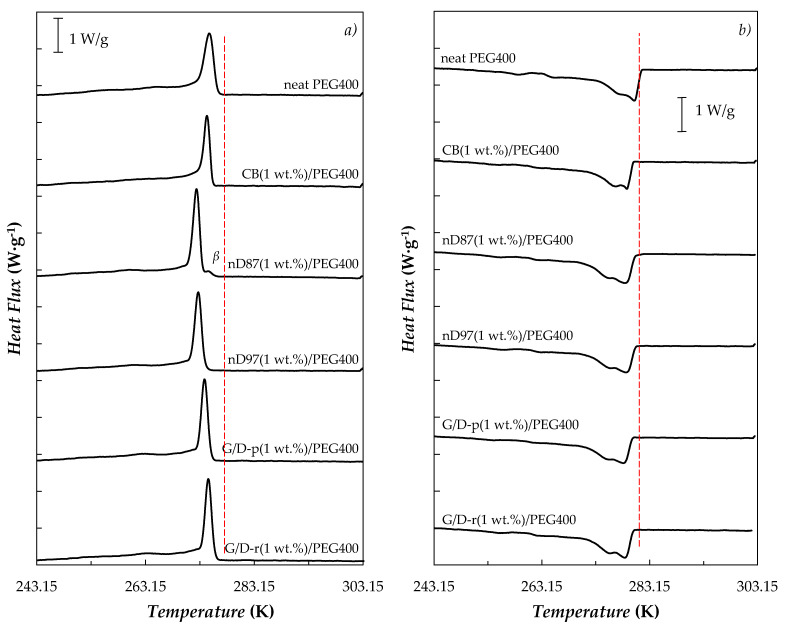
(**a**) Cooling and (**b**) heating differential scanning calorimetry (DSC) curves obtained at 0.5 K·min^−1^ for the neat PEG400 and the nanofluids prepared at 1 wt.% nanoparticle loadings.

Figure 2 shows the cooling and heating thermograms for neat PEG400 and the five dispersions prepared at the highest nanoparticle concentration (1.0 wt.%), while melting temperatures, crystallizing temperatures and melting enthalpies are presented in Table 2. Melting latent heats of neat PEG400 in the range from 105.3 to 117.3 J·g^−1^ are usually reported for poly(ethylene glycols) with similar molecular masses [11,73]. As can be observed, the addition of any of the five carbon nanostructures leads to shifts in melting and crystallizing transitions towards lower temperatures. This behavior was previously attributed in the literature [73,96] to the fact that the presence of the nanostructures reduces the mobility of poly(ethylene glycol) segments during solid-liquid change. As a consequence of the reduced ability of the polymer chains to bend/fold, PEG crystals formed during solidification are less thick and those thinner PEG crystals exhibit solid-liquid transitions at lower temperatures [97]. In addition, it is also possible to appreciate a reduction in the undesirable sub-cooling effect from 4.0 K (neat-PEG400) to 2.3 K (carbon black, CB, dispersion at 1.0 wt.%) or 2.0 K (raw graphite/diamond nanomixture, G/D-r, suspension at 1.0 wt.%). This is due to the improvement in the nucleation during crystallizing, as a consequence of the additional nucleation sites provided by dispersed nanostructures [76]. In addition, enthalpy of fusion decreases with the nanoparticle loading, with reductions ranging from 2.4% (nano-Diamonds purified with grade G01, nD97) to 6.9% (raw graphite/diamond nanomixture, G/D-r, suspension at 1.0 wt.%). These diminutions in the latent heat capacity can be attributed to a lower crystallinity degree of the polymer due to the presence of the nanomaterials. The crystallinity degree of a polymer, *X*_c_, can be quantified by means of the following expression [97]:

(1) $$X_{c}=100\cdot \frac{{\Delta h}_{\mathrm{melt}.}}{{\Delta h}_{\mathrm{melt}.}^{0}}$$

where $\Delta h_{\mathrm{melt}.}$ is the latent heat of fusion experimentally measured for the studied poly(ethylene glycol), while $\Delta h_{\mathrm{melt}.}^{0}$ is the latent heat corresponding to a polymer with 100% crystallinity. In this case a value of $\Delta h_{\mathrm{melt}.}^{0}$ = 196.8 J·g^−1^ was considered, as reported by Pielichowski et al. [97]. As presented in Table 2, studied neat poly(ethylene glycol) exhibits *X*_c_ = 54.3%, which reduces down to a value of 50.5% in the case of G/D-r(1 wt.%)/PEG400 NePCM. Crystallinity degrees here obtained for neat PEG400 by using Equation (1) are in the range from 53.5 to 59.7%, which could be calculated from the heats of fusions reported in the literature [11,73] for poly(ethylene glycol)s with similar molecular masses.

#### 3.1.2. Dynamic Oscillatory Rheological Analyses

Phase changes or internal structural readjustments in polymers are directly related to variations in their viscoelastic properties. Hence, dynamic rheological experiments were also used in order to obtain an alternative insight into the solid-liquid phase transitions of neat PEG400 and the carbon-based suspensions prepared at the highest nanoparticle content (1.0 wt.%). Similar studies have been performed for other novel materials based on poly(ethylene glycol) [98,99], alkanes/paraffins [98,100] or ionic liquids [101].

**Figure 3:**
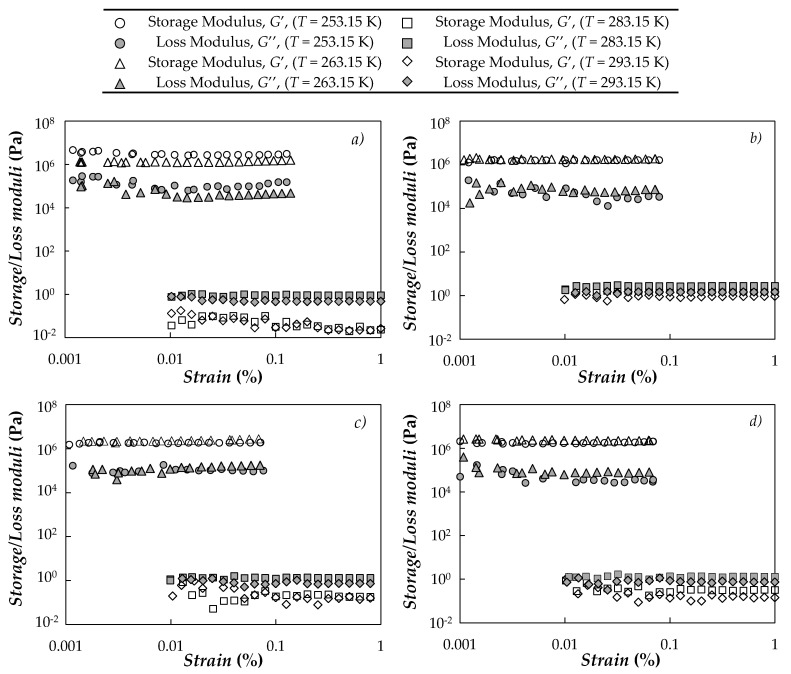
Storage and loss moduli as a function of strain obtained for (**a**) neat PEG400 as well as (**b**) CB (1 wt.%)/PEG400, (**c**) nD87 (1 wt.%)/PEG400 and (**d**) G/D-p (1 wt.%)/PEG400 nanofluids. Oscillatory frequency was kept constant at 1 Hz.

In order to partially define the linear viscoelastic region (LVR), strain sweeps were first performed at four temperatures: at 253.15 and 263.15 K (at which the PEG400 used as base fluid is in solid phase) as well as at 283.15 and 293.15 K (PEG400 is in liquid phase). As an example, Figure 3 shows the stain sweeps obtained at an angular frequency of 1 Hz for neat PEG400 and three representative dispersions (1 wt.% concentrations of either CB, nD87 or G/D-p).

As can be observed, in solid phase (see 253.15 and 263.15 K isotherms) dynamics storage, *G*′, and loss moduli, *G*″, remain nearly constant below oscillatory strains close to 1%. At those temperatures, *G*′ > *G*″ (at least one order of magnitude), evidencing the solid-like behavior of the samples. At 283.15 and 293.15 K isotherms, storage and loss moduli were almost constant for oscillatory strains larger than 0.01% (lower strains were not considered to avoid possible detection issues when working in liquid phase) while *G*″ > *G*′, which indicates that the base fluid and nanofluids present a liquid-like behavior at those temperatures.

**Figure 4:**
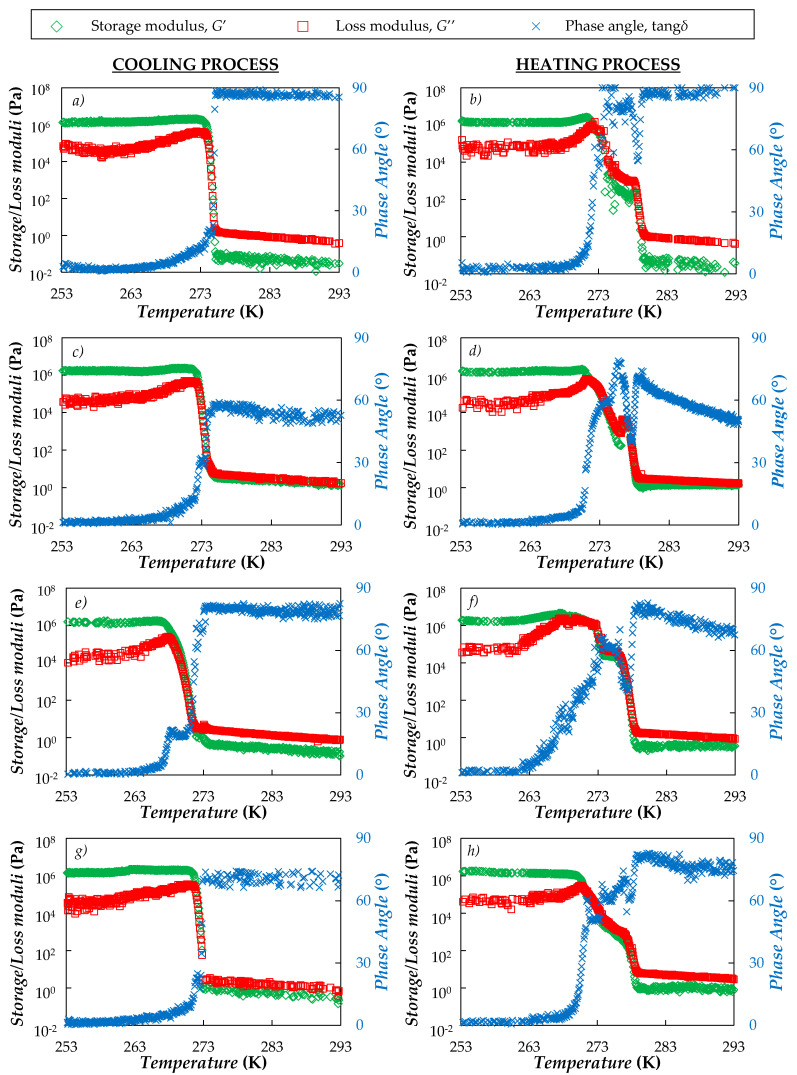
Temperature evolution of (◇) storage modulus, (□) loss modulus and (✕) loss phase angle obtained during (**a**,**c**,**e**,**g**) cooling and (**b**,**d**,**f**,**h**) heating processes at 0.5 K·min^−1^ for (**a**,**b**) neat PEG400, (**c**,**d**) CB(1 wt.%)/PEG400, (**e**,**f**) nD87(1 wt.%)/PEG400 and (**g**,**h**) G/D-p(1 wt.%)/PEG400. Test strain and frequency were kept constant at 0.05% and 1 Hz, respectively.

Then, the solid-liquid phase behavior was investigated throughout temperature sweeps in the range between 253.15 and 293.15 K, at constant cooling/heating rates of 0.5 K·min^−1^ (the same temperature scan conditions as those used in DSC analyses) and an oscillatory frequency of 1 Hz. In such experiments, oscillatory strain was set to 0.5% with the aim of ensuring that: (i) the experimental device was working in the region of optimal operating conditions and (ii) the material was being investigated within the linear viscoelastic range (LVR). Figure 4 shows the dynamic mechanical spectra (storage modulus, *G*′, loss modulus, *G*″, and damping factor, tang*δ*) as a function of temperature obtained for the neat PEG400 and three representative suspensions prepared at 1 wt.%.

In the cooling process of neat PEG400, at high temperatures the storage modulus is very low (*G*′ < 0.1 Pa) and at least one order of magnitude inferior to the loss modulus. When the temperature reaches ~275.8 K (close to the crystallizing point, *T*_cryst_ = 276.2 K, determined from DSC analyses) the storage modulus shows a sharp increase, while the damping factor sharply decreases from 86° to less than 10° (the crossover between *G*′ and *G*″, which indicates the beginning of the crystalline network formation, occurs at tang*δ* = 45°). As for the nanofluids, similar *G*′ and *G*″ evolutions were observed during the cooling processes. It must be pointed out that, even though nanofluids continue having a liquid-like appearance (in all cases tang*δ* > 45°) at temperatures at which the base fluid is supposed to be liquid, the addition of nanoparticles led to an increase in the storage modulus. This increase in the elastic behavior is usually observed when rigid elements are suspended in their network [102,103].

Heating curves show that the transition from solid to liquid-like phase occurs in a broader temperature range than crystallization. This is consistent with the DSC thermograms in which thermal events due to melting are prolonged from 263 to 280 K (Figure 2b). Additionally, in heating scans it is also possible to observe something similar to a plateau in *G*′ and *G*″. Such behavior was also reported in the literature for other materials based on poly(ethylene glycol)s or paraffin and agrees with the two superimposed peaks also observed in heating DSC thermograms (Figure 2b).

### 3.2. Dynamic Viscosity

**Figure 5:**
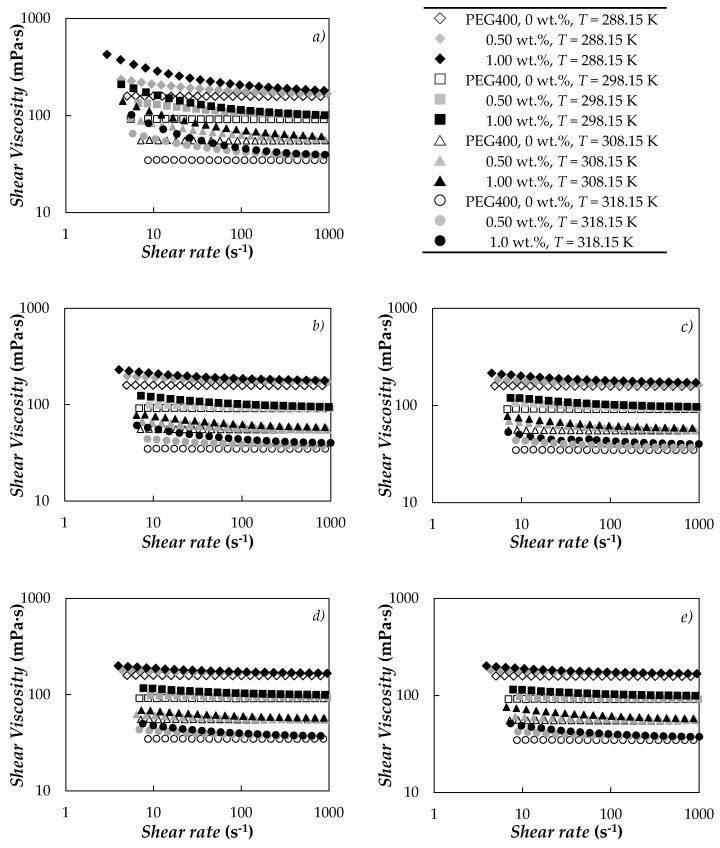
Flow viscosity curves of PEG400-based nanofluids containing: (**a**) CB, (**b**) nD87, (**c**) nD97, (**d**) G/D-p or (**e**) G/D-r carbon nanostructures.

The effect of particle loading on the shear rate-shear viscosity profile was studied for the neat PEG400 and the five carbon nanofluid sets by means of non-linear viscoelastic measurements. Figure 5 shows the steady shear viscosity curves obtained at the four studied temperatures. Dynamic viscosity results obtained for base PEG400 exhibit an absolute average deviation of 2.9% with the values reported by Marcos et al. [73] for a poly(ethylene glycol) with a similar molecular mass.

Unlike PEG400, the studied suspensions evidence a non-Newtonian behavior with a shear-thinning viscosity (i.e., dynamic viscosity reduces as shear rate increases), which gets stronger with the increasing nanoparticle content. Pseudo-plastic viscosity curves were also reported by Marcos et al. [71] when they studied the flow behavior of PEG400-based dispersions loaded with 0.05–1.0 wt.% of multi-walled carbon nanotubes. Żyła et al. [83,92] comprehensively investigated the rheological behavior of ethylene glycol dispersions containing nano-diamond mixtures (nD87 and nD97 [92]) as well as graphite/diamond mixtures (G/D-p and G/D-r [83]), analyzing the effect that nano-diamond purity and ash content have on shear rate-shear viscosity curves, among other thermophysical properties. Recently, Vallejo et al. [81] performed a complete rheological study of the dispersions of the same carbon-based nanomaterials used in the present study but based on ethylene glycol (EG). Vallejo et al. [81] reported a shear-thinning behavior for suspensions of these five carbon nanopowders (CB, nD87, nD97, G/D-p or G/D-r) in the nanoparticle concentration range from 0.25 to 2.0%. 

The non-Newtonian behavior of carbon-based PCMs can be described using the Ostwald-de Waele (power law) model, expressed as

(2) $$\eta =K\cdot {\dot{\gamma }}^{\;n-1}$$

where *K* and *n* fitting parameters are the flow consistency factor and the flow behavior index, respectively. Power law parameters obtained from the viscosity flow curves of the different studied suspensions at shear rates up to 100 s^−1^ are gathered in Table 3. A good description of the non-Newtonian region was observed for all studied dispersions, with absolute average deviations in dynamic viscosity equal to or lower than 4.2%. A comparison between the different studied nanofluid sets shows a much stronger shear-thinning degree in the case of carbon black (CB) dispersions, followed by nano-diamond sets (nD87 and nD97) and finally graphite/diamond nanomixtures (G/D-p and G/D-r). Thus, a minimum flow consistency index of *n* = 0.77 was observed at 318.15 K for the CB/PEG400 sample loaded with 1.0 wt.%, while the *n* value ranged from 0.87 to 0.99 for PEG400 dispersions prepared using the other carbon powders. When compared with EG dispersions prepared at equivalent concentrations of the same carbon-based nanopowders [81], PEG400 suspensions show a less strong pseudoplastic behavior. However, in both base fluids (EG [81] and PEG400), it is possible to observe that the dispersions of nano-diamonds (nD87 and nD97) and graphite/diamond nanomixtures (G/D-p and G/D-r) show a slightly stronger shear-thinning effect as the temperature increases.

The stronger pseudo-plastic behavior of carbon black suspensions can be attributed to the larger size and more irregular shape of those particles in comparison with the other four nanopowders. Small differences in the shear rate-shear viscosity curves when comparing the two nano-diamond sets (nD87 and nD97) or the two graphite/diamond nanomixture series (G/D-p and G/D-r) were attributed to different purity degrees or the ash content in the nanopowder by other authors [81,83,92] for studied ethylene glycol suspensions with higher concentrations of those same nanoadditives.

**Figure 6:**
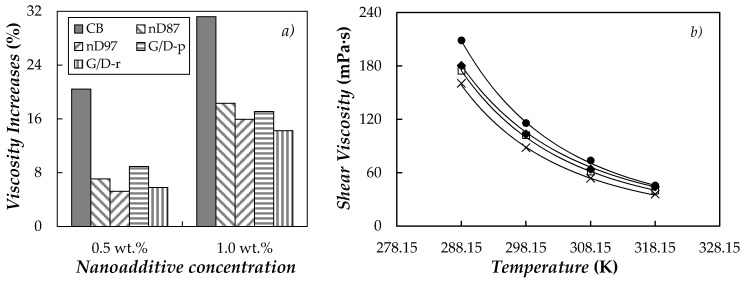
(**a**) Modifications in shear viscosity regarding neat PEG400 at ~100 s^−1^ and 298.15 K. (**b**) Temperature dependence of shear viscosity obtained at ~100 s^−1^ for neat PEG400 (✕) and nanofluids prepared at the highest nanoparticle concentration, 1.0 wt.%, of (⬤) CB, (□) nD87 and (◆) G/D-p. (Solid lines) Vogel-Fulcher-Tammann-Hesse, Equation (3).

As expected, nanofluid viscosity rises with the addition of nanoparticles. Figure 6a shows the increases in dynamic viscosity for the two studied concentrations and five carbon-based structures regarding the base PEG400. At that shear rate a higher viscosity is observed for CB/PEG400 samples than for the sets containing nano-diamonds (nD87 and nD97) or graphite/diamond nanomixtures (G/D-p and G/D-r).

Figure 6b shows the temperature dependence of the dynamic viscosities collected at a shear rate of ~100 s^−1^ for CB/PEG400, nD87/PEG400 and G/D-p/PEG400, as an example. In the whole analyzed temperature range (288.15–318.15 K), reductions in dynamic viscosity with increasing temperatures are ~75–79%. The characteristic downward trend in the viscosity of liquids with rising temperature, *η*(*T*), can be described by means of the following three-parameter logarithmic equation known as the Vogel-Fulcher-Tammann-Hesse [104,105,106] equation:

(3) $$\ln\eta (T)=\ln\eta_{0}+\frac{D\cdot T_{0}}{T-T_{0}}$$

where *η*_0_, *D* and *T*_0_ are the fitting coefficients, which provide physical information regarding the energy associated with packing of liquid molecules (*η*_0_), the dimensionless fragility of the liquid (1/*D*) and the ideal glass transition temperature (*T*_0_). The values of these coefficients as well as the *AAD%* and standard deviations between experimental values and data fitted by the Vogel-Fulcher-Tammann-Hesse model are gathered in Table 4.

Obtained experimental viscosities can be correlated with standard deviations in the range from 2.2 to 4.3 mPa·s (AADs% ~0.7–2.0%). Angell strength (*D*) values are in the range 6.16–6.46, which correspond to liquids with moderate to fragile behavior [107].

### 3.3. Thermal Conductivity

**Figure 7:**
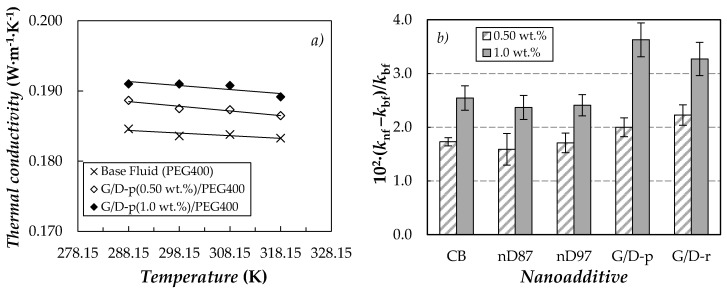
(**a**) Temperature dependence of thermal conductivity for (G/D-p)/PEG400 nanofluid system. (**b**) Average thermal conductivity enhancements regarding base PEG400, 10^2^·(*k*_nf_ − *k*_bf_)/*k*_nf_. Error bars represent the standard deviation between values obtained for the different studied temperatures.

Thermal conductivity, *k*, was studied for the base fluid and the ten carbon-based dispersions in the temperature range from 288 to 318 K. Results here obtained for neat PEG400 exhibit a good agreement (AADs% lower than 1.0%) with previous data measured by Marcos et al. [67,71,73] for poly(ethylene glycol) with similar molecular masses using other experimental devices based on the transient hot wire [71,73] or transient plane [67] methods. As an example, Figure 7a presents the temperature dependence of thermal conductivity for a PEG400 nanofluid set prepared with the purified graphite/diamond nanomixture (G/D-p).

As can be observed, the thermal conductivity of the phase change materials slightly improves with the addition of carbon-based nanoparticles. Improvements in this property reach 2.0% and 3.6% for the sample loaded with 0.5% and 1.0% of G/D-p, respectively (see Figure 7b). The enhancements in thermal conductivity presented here are lower than the increases of 12.7% and 23% reported for PEG400-based nanofluids containing 1 wt.% of MWCNTs [71] or 0.50 wt.% of functionalized graphene nanoplatelets [73], respectively. Nevertheless, our *k* modifications are similar to the maximum enhancement of 3.9% reported for a 1.1 wt.% suspension of PVP-capped silver nanoparticles in PEG400 [67]. 

### 3.4. Volumetric Behavior

**Figure 8:**
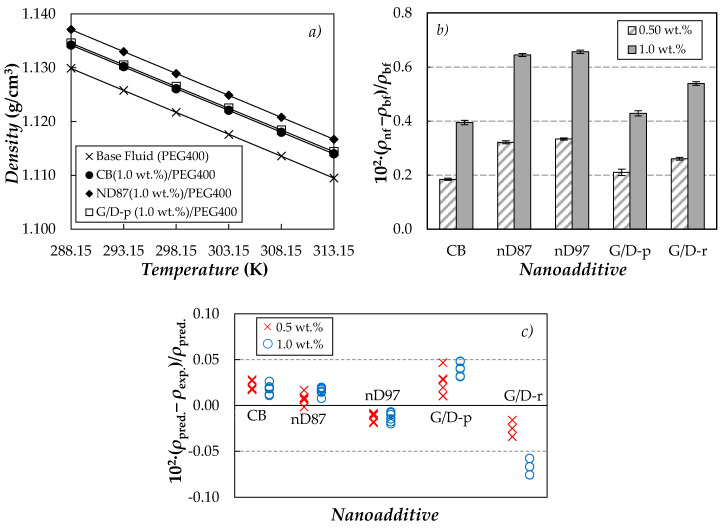
(**a**) Temperature dependence of density for neat PEG400 and nanofluids prepared at the highest nanoparticle concentration, 1.0 wt.%, of (⬤) CB, (□) nD87 and (◆) G/D-p. (**b**) Average density modifications regarding base PEG400, 10^2^·(*ρ*_nf_ − *ρ*_bf_)/*ρ*_nf_. Error bars represent the standard deviation between values obtained for the different studied temperatures. (**c**) Deviations between experimental densities, *ρ*_exp._, and values predicted using Equation (4), *ρ*_pred_.

Densities, *ρ*, of neat PEG400 and the five carbon-based suspensions were experimentally measured at atmospheric pressure and in the temperature range from 288.15 to 313.15 K. As an example, experimental results obtained for the base fluid and the dispersions prepared with the highest concentration (1.0 wt.%) of either carbon black (CB), nano-diamonds purified with grade G (nD87) or purified graphite/diamond nanomixture (G/D-p) are presented as a function of temperature in Figure 8a.

Maximum deviations lower than 0.05% were observed when the densities obtained for PEG400 in the framework of this investigation were compared with results reported in the literature [73,108,109,110] for poly(ethylene glycols) with similar molecular mass. Density increases with the increasing nanoparticle concentration, without any temperature effect on such enhancements. Average density modifications (regarding neat PEG400) obtained for the five different carbon-based nanofluid sets are plotted in Figure 8b. Higher *ρ* enhancements were observed for suspensions containing purified nano-diamonds (nD87 and nD97) than for the rest of carbon-based nanostructures. This result can be attributed to the higher density of nano-diamonds (usually in the range of 3.1–3.3 g·cm^−3^ [84]), when compared to graphite or carbon black (1.9–2.3 g·cm^−3^ reported elsewhere [82]). Modifications presented here are in parallel with the maximum enhancement of 0.33% reported for a 0.5 wt.% (graphene nanoplatelet)/PEG dispersion by Marcos et al. [73] or the increase of 0.42% obtained for MWCNT(1 wt.%)/PEG400 in [71]. A good agreement was also found between density modifications here reported for nD87/PEG400 and nD97/PEG400 and those previous measured for nD87/EG and nD97/EG suspensions by Żyła et al. [92]. Experimental *ρ* measured in this work for PEG400-based suspensions were also compared with the values calculated using the following weight-average equation:

(4) $$\frac{1}{\rho_{\mathrm{nf}}}=\frac{\varphi }{\rho_{\mathrm{np}}}-\frac{1-\varphi }{\rho_{\mathrm{bf}}}$$

where *φ* is the mass fraction of the nanoparticles, while the subscripts nf, bf and np stand for nanofluid, base fluid and nanoparticles, respectively. In this case, nanoparticle densities of 1.9, 3.1, 3.2, 2.52 and 2.54 g/cm^−3^ were considered for carbon back (CB) [82], nano-diamonds purified with grade G (nD87), nano-diamonds purified with grade G01 (nD97), purified graphite/diamond nanomixture, (G/D-p) [83] and raw graphite/diamond nanomixture (G/D-r) [83], respectively. The densities for the two nano-diamond mixtures were calculated as the weight average value between the densities of graphite (2.26 g/cm^−3^ [82]) and nano-diamonds (3.2 g·cm^−3^ [84]), considering the amount of nano-diamonds (87 and 97% in mass) and impurities (all assumed with the same density as graphite). As shown in Figure 8c, deviations between experimental densities and values predicted using Equation (4) are within 0.1%.

Like for the base fluid, the nanofluid densities decrease as temperature increases. This *ρ*(*T*)-dependence can be correlated with AADs% lower than 0.001% by means of second-order polynomial fittings. Isobaric thermal expansivities, *α*_p_ = −(1/*ρ*)(∂*ρ*/∂*T*)_p_, can be numerically calculated from the derivatives of those polynomial density adjustments. At the studied conditions, the obtained values are in the range (7.15–7.45) × 10^−4^·K^−1^. This property mainly reduces with the nanoparticle loading, with maximum diminutions reaching 1.2% and 1.4% for CB (1.0 wt.%)/PEG400 and G/D-r (1.0 wt.%)/PEG400 samples, respectively. In comparison with liquids, solid carbon materials are well known to show high elastic stiffness when subject to changes in temperature. Thus, at room temperature single crystal diamond exhibits an isobaric thermal expansion coefficient around (7 ± 3) × 10^−7^·K^−1^, for example. According to a weight rule mixture based on the volume fraction of the nanoparticles, i.e., *α*_p,nf_ *= ϕ·α*_p,np_ + (1 − *ϕ*)·*α*_p,bf_, the addition of carbon-based nanostructures is expected to reduce apparent thermal conductivity. Such behavior was observed in the literature for graphene dispersions into ethylene glycol-water mixtures [73,111] or poly(ethylene glycol) [112], for instance. However, dissimilar results were also reported. Thus, some authors [103,113] observed reductions in the isobaric heat capacity when adding nanoparticles. Such changes may be attributed to a minor degree of cohesion in relation to base fluids, due to the presence of surfactant molecules or possible interactions between base fluid and solid nanoparticles.

### 3.5. Surface Tension

**Figure 9:**
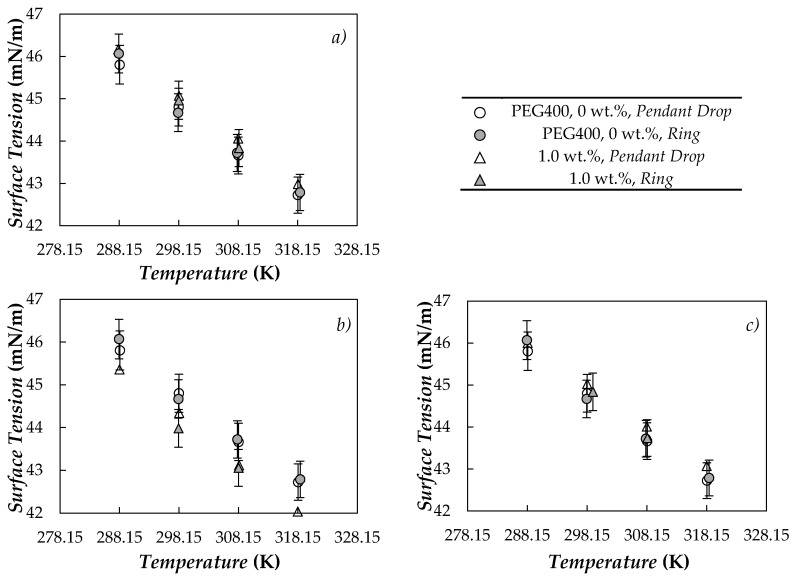
Temperature dependence of surface tension obtained for neat PEG400 and nanofluids prepared at the highest nanoparticle concentration, 1 wt.%, of (**a**) CB, (**b**) nD87 and (**c**) G/D-p.

Surface tension, SFT or *σ*, measurements of base PEG400 and the ten nano-dispersions were performed in the temperature range from 288 to 318 K by means of the DSA-30 drop-shape analyzer. Following the investigation recently published by Wanic et al. [93], experimental STFs were also determined at 298 and 308 K for samples loaded with 1.0 wt.% using a PI–MT1A.KOM Du Noüy ring surface tensiometer. As an example, Figure 9 shows the temperature dependence of surface tension for the base fluid and nanofluids containing 1.0% concentrations of either carbon black (CB), nano-diamonds purified with grade G (nD87) or purified graphite/diamond nanomixture (G/D-p).

**Figure 10:**
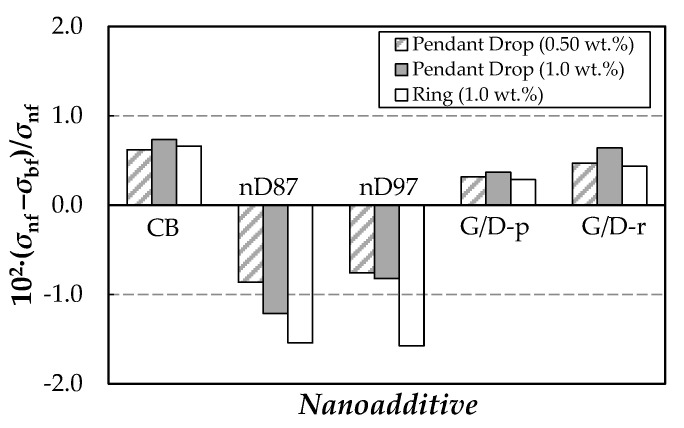
Average modifications in surface tension regarding pure PEG400, 10^2^·(*σ*_nf_ − *σ*_bf_)/*σ*_nf_, obtained for the 0.50 and 1.0 wt.% nanofluids using the pendant drop method and for 1.0 wt.% nanofluids using the ring method.

A good agreement was found between the experimental results determined in this investigation for the base fluid (PEG400) and previous SFTs reported for poly(ethylene glycol)s with close molecular mass by Fu et al. [114] or Marcos et al. [67]. A comparison between the values provided for both base PEG400 and 1.0 wt.% samples by Du Noüy ring and drop-shape surface tensiometers shows deviations lower than or equal to 0.9%, which is well within the combined experimental uncertainty of both devices. Carbon-based suspensions present the same downward trend with increasing temperature as exhibited by the neat PEG400. In the studied interval (288–318 K), SFT reductions with rising temperature are 2.1–2.7% each 10 K. Regarding the nanoparticle loading effect on SFT, different behaviors can be observed depending on the carbon nanostructure. Average modifications in surface tension (regarding the base fluid) for the different samples are presented in Figure 10.

Reductions in SFT were obtained for the two nano-diamond nanopowders (nD87 and nD97), while slight increases were observed for dispersions prepared using the other three carbon-based nanopowders. However, it must be pointed out that, except for results obtained for 1.0% concentrations of nD87/PEG and nD97/PEG sets using the Du Noüy ring method and 1.0% loading of nD87/PEG using the drop-shape analyzer, all modifications are within the experimental uncertainties reported by these devices 1.0% [93]. 

## 4. Conclusions

In this paper, phase change materials based on PEG400 and containing five different carbon-based materials (viz., a carbon black nanopowder, two graphite/diamond nanomixtures and two nano-diamond nanopowders with two different purity grades) are thermally and physically characterized as potential phase change materials to cold energy storage media. First, mechanical and thermal changes occurring during the solid-liquid phase transition were analyzed throughout differential scanning calorimetry and oscillatory rheology studies. The dispersion of nanoparticles reduced the undesirable sub-cooling effect from 4.0 K (neat-PEG400) to 2.3 K (carbon black dispersion at 1.0 wt.%) or 2.0 K (raw graphite/diamond nanomixture suspension at 1.0 wt.%). A good agreement (less than 0.8 K) was observed between the crystallizing temperatures determined using DSC and detected during dynamic oscillatory rheological tests. The effects of particle loading (0.5 wt.% and 1.0 wt.%) and carbon nanostructure type (CB, nD87, nD97, G/D-p and G/D-r) on dynamic viscosity, thermal conductivity, density and surface tension were also investigated in the temperature range from 288.15 to 318.15 K. According to the non-linear rheological experiments, all studied suspensions showed a non-Newtonian pseudo-plastic behavior. Shear-thinning degree rose with increasing nanoparticle loading and was stronger in the case of carbon black (CB) dispersions, followed by nano-diamond sets (nD87 and nD97) and finally graphite/diamond nano-mixtures (G/D-p and G/D-r). Larger thermal conductivity improvements were observed in the case of suspensions containing purified (3.6%) and raw (3.3%) graphite/diamond nanomixtures, while nD87 and nD97 nano-diamond suspensions showed the maximum modifications in density (increases of up to 0.64–0.66%). A good agreement was observed between surface tension measurements performed using drop-shape analyzer and Du Noüy ring methods. Reductions in this property were observed for the two nano-diamond nanopowders (nD87 and nD97), while slight increases (within experimental uncertainties) were observed for dispersions prepared using the other three carbon-based nanopowders. Analyzing the effect that the chemical functionalization of the nanoparticles could have on the phase change characteristics and thermal behavior of the nano-enhanced phase change materials should be considered in future works. Additionally, non-dimensional analyses or economic studies could help to optimize the most appropriate concentration of nanoparticles.

## Figures and Tables

**Table 1:** Studied carbon nanopowders ^†^.

Label in the Article	Nanopowder Description	Average Particle Size (nm)	Diamond Content	Non-Diamond Carbon Content	Ash Content
CB	carbon black	13	-	-	<0.02 wt.%
nD87	nano-diamonds, purified, grade G	4	>87 wt.%	<5–7 wt.%	<6 wt.%
nD97	nano-diamonds, purified, grade G01	4	>97 wt.%	<0.7 wt.%	<1.4 wt.%
G/D-p	graphite/diamond nanomixture, purified	4	>20 wt.%	-	<0.3 wt.%
G/D-r	graphite/diamond nanomixture, raw	4	>20 wt.%	-	<6 wt.%

^†^ Information supplied by the manufacturer (PlasmaChem GmbH, Berlin, Germany).

**Table 2:** Crystallizing, *T*_cryst._, and melting, *T*_melt._, temperatures; latent heat of fusion, Δ*H*_melt._, and crystallinity degrees, *X*_c_, obtained for neat PEG400 and dispersions prepared at 1 wt.% nanoparticle concentrations from DSC scans at 0.5 K·min^−1^.

Sample	*T*_cryst._ (K)	*T*_melt._ (K)	Δ*H*_melt. (_J·g^−1^)	*X*_c_
neat PEG400	276.2	280.2	106.8	54.3
CB(1 wt.%)/PEG400	276.0	278.2	101.2	51.4
nD87(1 wt.%)/PEG400	275.9 (*β*), 273.8	278.0	99.6	50.6
nD97(1 wt.%)/PEG400	274.3	278.5	104.2	52.9
G/D-p(1 wt.%)/PEG400	275.4	278.0	100.3	51.0
G/D-r(1 wt.%)/PEG400	276.0	278.0	99.4	50.5

**Table 3:** Flow consistency factor (*n*), flow behavior index (*K*) and absolute average deviations (AAD%) obtained from the Ostwald-de Waele equation, Equation (2), modelling the shear-thinning region up to 100 s^−1^ of the different studied dispersions.

Parameter	CB		nD87		nD97		G/D-p		G/D-r	
	0.5 wt.%	1.0 wt.%	0.5 wt.%	1.0 wt.%	0.5 wt.%	1.0 wt.%	0.5 wt.%	1.0 wt.%	0.5 wt.%	1.0 wt.%
	***T* = 288.15 K**									
***K* (mPa·s^n^)**	262.91	480.82	203.49	243.45	191.56	225.13	186.03	211.33	186.64	213.41
***n***	0.91	0.80	0.97	0.95	0.97	0.95	0.98	0.95	0.98	0.95
**AAD%**	0.8	2.2	0.3	1.3	0.4	0.6	0.1	0.4	0.2	0.3
	***T* = 298.15 K**									
***K* (mPa·s^n^)**	161.13	269.64	106.86	142.99	116.65	135.98	100.07	128.66	101.59	127.45
***n***	0.91	0.79	0.97	0.94	0.96	0.94	0.99	0.95	0.98	0.95
**AAD%**	0.6	2.2	0.1	0.4	0.3	0.6	0.2	0.3	0.1	0.4
	***T* = 308.15 K**									
***K* (mPa·s^n^)**	100.41	178.29	72.05	82.30	76.82	89.73	64.94	75.82	63.46	76.82
***n***	0.90	0.78	0.97	0.92	0.94	0.92	0.96	0.95	0.97	0.94
**AAD%**	0.7	2.2	0.4	0.1	0.4	0.8	0.9	0.3	0.1	0.3
	***T* = 318.15 K**									
***K* (mPa·s^n^)**	72.81	130.74	63.46	72.05	48.84	63.02	46.46	56.77	46.59	57.64
***n***	0.88	0.77	0.95	0.87	0.95	0.91	0.95	0.92	0.96	0.92
**AAD%**	1.1	4.2	0.4	1.1	0.5	3.8	1.4	0.9	0.4	1.0

**Table 4:** Fitting parameters (*η*_0_, *D*, *T*_0_), absolute average deviations (*AAD%*) and standard deviation (*s*) obtained from the Vogel-Fulcher-Tammann-Hesse model, Equation (3), when modelling the temperature dependence of shear viscosity obtained at ~100 s^−1^ for neat PEG400 and carbon-based PEG400 dispersions.

Set	Nanoparticle/wt.%	Fitting Parameters			AAD%	*s*/mPa·s
		*η*_0_/mPa·s	*D*	*T*_0_/K		
PEG400	0	0.0556	6.23	161.68	1.3	2.2
CB	0.5	0.0773	6.37	158.16	1.1	3.7
nD87	0.5	0.0580	6.37	158.16	1.3	3.5
nD97	0.5	0.0724	6.26	161.15	2.0	4.3
G/D-p	0.5	0.0718	6.35	161.15	0.7	2.9
G/D-r	0.5	0.0718	6.35	161.15	1.2	3.0
CB	1.0	0.0886	6.46	161.15	1.5	3.4
nD87	1.0	0.0551	6.19	158.16	1.1	3.7
nD97	1.0	0.0553	6.16	158.16	1.5	4.0
G/D-p	1.0	0.0572	6.18	158.16	2.0	3.0
G/D-r	1.0	0.0748	6.25	161.15	0.7	2.9

## Nomenclature

AAD%	Absolute average deviation
*α*_p_	Isobaric thermal expansivity (K^−1^)
CB	Carbon black
*D*, *η*_0_, *T*_0_	Vogel-Fulcher-Tammann-Hesse fitting coefficients
DSC	Differential scanning calorimetry
EG	Ethylene glycol
G/D-p	Purified graphite/diamond nanomixture
G/D-r	Raw graphite/diamond nanomixture
*k*	Thermal conductivity (W·m^−1^·K^−1^)
*K*	Flow consistency factor (mPa·s^n^)
MWCNT	Multiwalled carbon nanotubes
*n*	Flow behavior index
nD87	Nano-diamonds, purified, grade G
nD97	Nano-diamonds, purified, grade G01
NePCM	Nano-enhanced phase change material
PCM	Phase change material
PEG	Poly(ethylene glycol)
*T*	Temperature (K)
TES	Thermal energy storage
*β*	Heating/cooling rate (K·min^−1^)
Δ*H*	Latent heat (J·g^−1^)
*η*	Dynamic viscosity (mPa·s)
*ρ*	Density (g·cm^−3^)
*σ*	Surface tension, SFT, (mN·m)
*φ*	Mass fraction
*ϕ*	Volume fraction
nD97	Nano-diamonds, purified, grade G01
G/D-p	Purified graphite/diamond nanomixture
G/D-r	Raw graphite/diamond nanomixture.
*X*_c_	Crystallinity degree
*p*	Pressure (MPa)
*s*	Standard deviation
wt.%	Mass concentration
**Subscripts**	
bf	Base fluid
cryst.	Crystallization
melt.	Melting
nf	Nanofluid
np	Nanoparticle
**Superscript**	
0	100% crystallinity
